# Elaboration of a new framework for fine-grained epidemiological annotation

**DOI:** 10.1038/s41597-022-01743-2

**Published:** 2022-10-26

**Authors:** Sarah Valentin, Elena Arsevska, Aline Vilain, Valérie De Waele, Renaud Lancelot, Mathieu Roche

**Affiliations:** 1 UMR TETIS (Land, Environment, Remote Sensing and Spatial Information), University of Montpellier, AgroParisTech, CIRAD, CNRS, INRAE, Montpellier, France; 2 UMR ASTRE (Unit for Animals, Health, Territories, Risks and Ecosystems), University of Montpellier, CIRAD, INRAE, Montpellier, France; 3 Department of Biology, University of Sherbrooke, Sherbrooke, Canada; 4 Quebec Centre for Biodiversity Science, McGill University, Montreal, Canada; 5 French Agricultural Research for Development (CIRAD), Montpellier, France; 6 Veterinary Epidemiology Service, Departement of Epidemiology and Public Health, Sciensano, Brussels, Belgium; 7 Department of Environmental and Agricultural Studies, Public Service of Wallonia, B5030 Gembloux, Belgium

**Keywords:** Research data, Agriculture

## Abstract

Event-based surveillance (EBS) gathers information from a variety of data sources, including online news articles. Unlike the data from formal reporting, the EBS data are not structured, and their interpretation can overwhelm epidemic intelligence (EI) capacities in terms of available human resources. Therefore, diverse EBS systems that automatically process (all or part of) the acquired nonstructured data from online news articles have been developed. These EBS systems (e.g., GPHIN, HealthMap, MedISys, ProMED, PADI-web) can use annotated data to improve the surveillance systems. This paper describes a framework for the annotation of epidemiological information in animal disease-related news articles. We provide annotation guidelines that are generic and applicable to both animal and zoonotic infectious diseases, regardless of the pathogen involved or its mode of transmission (e.g., vector-borne, airborne, by contact). The framework relies on the successive annotation of all the sentences from a news article. The annotator evaluates the sentences in a specific epidemiological context, corresponding to the publication date of the news article.

## Abstract (summary)

**Table:** 

Measurement(s)	Precision/Recall • Annotator aggreement
Technology Type(s)	Expert annotation • Kappa’s coefficient • Natural Language Processing
Sample Characteristic - Organism	Animal • African swine fever virus • Avian influenza • Foot-and-mouth disease virus • Bluetongue virus • Bovine spongiform encephalopathy
Sample Characteristic - Environment	Animal infectious diseases

## Background & Summary

In this paper, we first describe the needs for developing a new annotation framework by highlighting the limitations of the current approaches and available resources. We further describe our global protocol for guideline elaboration, followed by a detailed description of the final annotation guidelines. We discuss how we address the annotation challenges of the global process, and we highlight the contributions and limitations of our framework.

**Fig. 1:**
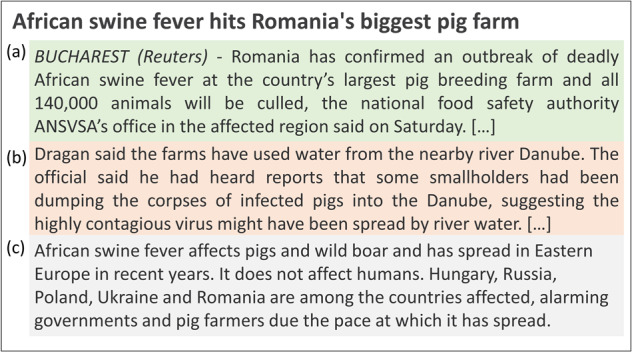
Extract of a news article published by Reuters on August 25, 2018. This news describes an African swine fever outbreak in Romania including the outbreak description (**a**), the transmission pathway of the outbreak (**b**) and general epidemiological knowledge about the disease and its spread (**c**). The whole news content is available at: journal https://www.reuters.com/article/us-romania-swineflu-pigs-idUSKCN1LA0LR.

Classification in text mining usually assigns a single topic (category) per news item (document-based classification). However, animal health news is rich in different types of epidemiological information. For instance, news articles that report an outbreak often also describe the outbreak control measures or economic impacts and point to the outbreak source or area at risk (Fig. 1). These elements may be of relevance to epidemic intelligence (EI) teams to assess the risks associated with the occurrence of a disease outbreak (further referred to as an event).

When the news contains several topics, a single-label classifier has to decide on a topic (i.e., a label) among several possible topics, usually decreasing the classification performance^1^. Most classification approaches in EBS systems focus on binary news relevance. Little attention has been focused on the retrieval of other types of epidemiological information.

In this context, we propose to split news content into sentences that are annotated into different categories according to their epidemiological topic, which we refer to as fine-grained information. Empirically, sentence-level classification seems more homogeneous in terms of topics than document-level classification. We therefore believe that sentence-level classification can more accurately identify specific types of information.

To create annotated data as part of a machine learning pipeline useful for EI practitioners, we first need to elaborate and evaluate a generic annotation framework that should be as reproducible as possible. In addition, the list of classes for sentence-based annotation should allow us to identify new types of epidemiological information in animal disease-related news.

## Methods

### Related work

Supervised learning algorithms implemented in EBS systems must be trained on labelled datasets to further classify unknown data. Several annotated textual resources thus have been created to support classifier training tasks in animal health. Table 1 presents examples of labelled datasets of news in the animal disease surveillance context. Datasets are compared based on their aim, the characteristics of the annotated data and their reproducibility in terms of availability (indicating whether the corpus and guidelines are freely available for download) and reliability (corresponding to the evaluation of inter-annotator agreement).

**Table 1:** Example of annotated data used for online news processing in event-based surveillance applications. C: classification, NER: named entity recognition, EE: event extraction.

Data source	Tasks	Annotated data				Annotation guidelines		Limitations
		Content	Annotation unit	Labels	Availability	Agreement evaluation	Availability	
BioCaster	C	Corpus of 1,000 news articles	Document	Alert, publish, check, reject	No	No	Yes (brief)	Document-based, Corpus unavailable, Reproducibility
FMD BioPortal	C	Corpus of 1,674 news articles	Document	Outbreak-related, control program-related, general information	No	No	Yes (brief)	Document-based, Corpus unavailable, Reproducibility
Argus	C	Corpus of news articles	Document	Relevant: yes/no	No	Yes	Yes (brief)	Corpus unavailable
ProMED	C	Corpus of 2,342 sentences	Sentence	Disease report: yes/no	No	No	No	Corpus unavailable, Reproducibility
PADI-web	C	Corpus of 600 news articles	Document	Relevant: yes/no	No	No	Yes (brief)	Document-based, Corpus unavailable, Reproducibility
	NER	Corpus of 532 news articles	Entity	Location, date, disease, host, number of cases	Yes	No	Yes (brief)	Entity-based, Reproducibility
BioCaster	NER	Multilingual ontology	Entity	Location, organisation, date, etc.	Yes	No	Yes (detailed)	Entity-based, Reproducibility
	EE	Corpus of 200 news articles	Event (disease-location pairs)	Event attributes: location, date, host, etc.	Partial	Yes	Yes (brief)	Event-based, Corpus partially unavailable
	EE	Corpus of 100 news articles	Event (a verb and a subject)	Event labels: temporally-locatable, generic, hypothetical. Event attributes: date, location, etc.	No	Yes	Yes (detailed)	Event-based, Corpus partially unavailable

Depending on the context in which it was created (typically the scope of the EBS system), the labelled corpus is either generalist, i.e. encompassing both human and animal disease events^2,3^, or specific, i.e. targeting one or several animal diseases^1^. The annotation unit and labels (categories) closely depend on the aim of the text-mining tasks in the animal disease domain, i.e. (i) classification, (ii) named entity recognition and (iii) event extraction.

(i). For classification tasks, annotation is usually at the document level. The labels are often related to the news relevance so as to filter out irrelevant ones^4–7^. Other classification frames assign a broad thematic label to the news, such as “outbreak-related” or “socioeconomic”^1^. To our knowledge, all document-based annotation approaches allow a single label per news piece.
(ii). For named entity recognition tasks, the corpus is annotated at the word level (including multi-word expressions). A typical example is the annotation framework of the BioCaster Ontology^2^.
(iii). For event extraction tasks, the annotation unit depends on the definition adopted for the event. Some authors opt for a linguistic definition, i.e. a verb (called predicate) and a subject or object (called argument). Some sophisticated event annotation schemes allow extraction of fine-grained temporal information such as the beginning and end of an event^2^, or thematic attributes such as the transmission mode^3^.

No currently available annotated data and frameworks can fulfil the needs of our current objective to detect fine-grained epidemiological information (i.e., topics). Document-based approaches are not precise enough to detect the variety of information contained in news articles. Word-based annotation frameworks provide accurate information at the word level, yet they are task-oriented (extraction of events or named entities) and partly address the potential of other types of epidemiological information. Midway between these two approaches^8^, proposed a sentence-based annotation to detect outbreak-related sentences, while recognising that a news piece contains many sentences with different semantic meanings. However, as the primary goal was outbreak detection, outbreak-unrelated sentences (e.g., describing treatment or prevention) were all merged into one negative category.

In addition to the shortcomings of the works mentioned above, the availability and reproducibility of the annotated data and guidelines vary between the studies. Several corpora were not published or are no longer available because of unstable storage. For instance, the BioCaster disease event corpus has to be retrieved through a Perl script that downloads documents from their web source. As some sources become unavailable, the corpus size inevitably decreases over time (only 102 source web pages among 200 were still available online in 2015^9^). The availability of EBS systems also hampers data access–two EBS systems from Table 1 were no longer operational (Argus, BioCaster).

Most of the proposed approaches lack reproducibility. First, annotation guidelines usually consist of brief label descriptions rather than detailed schemes. Second, in the provided examples, only three annotation frameworks were evaluated in terms of inter-annotator agreement. According to biomedical text annotation recommendations^10^, the BioCaster disease event corpus authors used percentage scores (pairwise agreement) rather than the kappa statistic^3^. The annotation framework of BioCaster Ontology included both metrics^2^. Multi-kappa statistics is also used to take into account more than two annotators for agreement measurement^6,11^.

Similarly to the approach of Zhang *et al*.^8^, we aim at implementing sentence-level annotation to enrich the binary outbreak-related/unrelated classification with thematic categories. Our objective is to make effective use of the epidemiological information contained in the news, especially when the information is relevant for assessing an epidemiological situation.

### Our approach

In this section, we describe our approach to building annotated resources for the extraction of fine-grained epidemiological information. We first describe the global process we adopted to develop the annotation guidelines. Then we present the final annotation framework and describe the proposed categories (labels). The annotation guidelines and annotated corpus are publicly available^12^.

This dataset is used in the context of the PADI-web (Platform for Automated extraction of Disease Information from the web) system. Briefly, PADI-web is an automated system devoted to online news source monitoring for the detection of animal infectious diseases. The tool automatically collects news via customized multilingual queries, classifies them using machine learning approaches and extracts epidemiological information (e.g., locations, dates, hosts, symptoms, etc.) with Natural Language Processing (NLP) approaches. In^13^ we summarized how the corpus described in this study is used to learn a fine-grained classification model based on a machine learning approach that is integrated into PADI-web 3.0. The proposed annotation scheme is intended to enhance EBS systems by enabling the automated classification of sentences from disease-related news. One of the main applications is the enhancement of the performances of event-extraction tasks by identifying the event-related sentences. We believe that performing event extraction on a subset of relevant sentences would decrease the risk of extracting epidemiological entities (e.g., dates, locations) not related to an event. In addition, the distinction between current and risk events allows for characterizing an event as ongoing or likely to occur. Sentences related to transmission pathways could be manually or automatically compared to current disease knowledge to identify the emergence of a new transmission pathway. Eventually, sentence-based classification is an alternative approach to increase the performance of document-based classification, especially in the context where event-related information appears within a few sentences^14^: each sentence can be first classified as relevant or not, and the results of each sentence classification can be merged to classify the document.

#### Global annotation process

We extracted 32 candidate English news items from the PADI-web database, while focusing on those classified as relevant. By relevant news, we mean a news report that is related to a disease event (describing a current outbreak as well as the prevention and control measures, preparedness, etc.). The classification in PADI-web is performed each day automatically and relies on a supervised machine learning approach; a family of classifiers is trained on a corpus of 600 news items manually labeled by an epidemiology expert (200 relevant news articles and 400 irrelevant news).

**Fig. 2:**
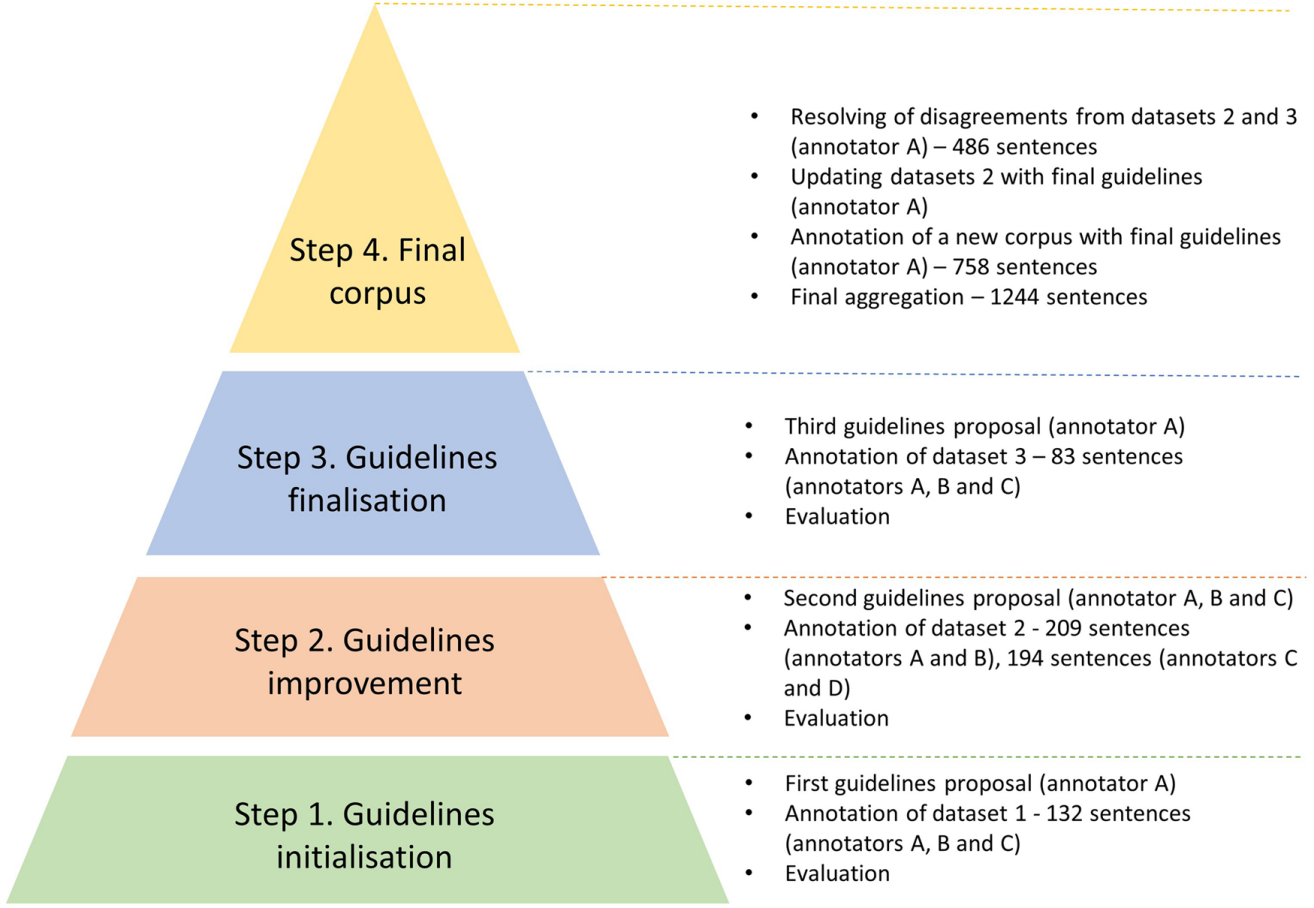
Pipeline of the annotation guideline elaboration process.

The four annotators (A, B, C, D) were veterinarians working in epidemic intelligence. Two of them had previous experience with annotation tasks (annotators A and B). During the process, we followed the four consecutive steps detailed in Fig. 2. After each annotation step, we calculated the agreement metrics.

The annotators discussed the main disagreement results and modified the guidelines to improve the annotation process. We describe the main modification choices that led to the final guidelines. We stopped the process when satisfactory agreement measures were attained, i.e., when the overall agreement was above 80% (Step 3).

To build the final corpus (step 4), we aggregated datasets 2 and 3. To choose one label per sentence in case of disagreement, we adopted the following procedure:

1. For dataset 3 (labelled by three annotators):
  - If at least two out of three annotators assigned the same label, we selected the majority label,
  - If each of the three annotators assigned a different label, annotator A chose a final label among those proposed;
2. For dataset 2 (labelled by two annotators):
  - If both annotators disagreed, annotator A chose a final label among those proposed,
  - Annotator A verified consistency with the final guidelines.

The corpus was further increased by annotating a new dataset with the final guidelines (see section Data Records).

#### Annotation guidelines

In this subsection, we present the final annotation framework and definitions of each label from the guidelines. A detailed version of this framework and the labelled corpus are publicly available in a Dataverse repository^12^.

**Fig. 3:**
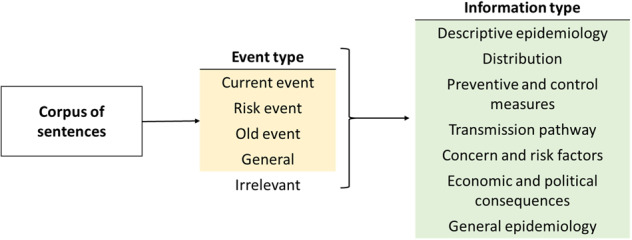
Two-level annotation framework.

In our framework, each sentence is annotated with one label for event type and one label for information type, as illustrated in Fig. 3. The event type level identifies if the sentence is related to an outbreak event, and, if so, the temporal relation with the event. The information type level describes the type of epidemiological information, i.e. the fine-grained topics. The meaning of a sentence depends on the entire news content, as well as its epidemiological context. Therefore, for each set of sentences (from a single news), the annotator first reads the news metadata (i.e. title, source, and publication date). The annotator chooses a single label per level and per sentence. As some sentences may contain information belonging to several information type categories, the annotator must pinpoint the primary information.

##### Event type

While focusing on sentence epidemiological topics, the relation between the sentence and the current epidemiological situation must be taken into account: sentences in news pieces may describe an outbreak that happened several years before or provide general information about a disease. More precisely, from the EBS standpoint, only sentences referring to current events or events at risk are of interest.

In our context, we define an event as the occurrence of a disease within a specific area and time range. The event type label aims to differentiate sentences referring to the current/recent outbreak (“Current event” and “Risk event”) from sentences referring to old outbreaks (“Old event”) or general information (“General”). Sentences which do not contain any epidemiological information are considered irrelevant (“Irrelevant”).

- **Current event**: this class includes sentences related to the current situation. There are five major groups of sentences that are considered “current”:
  1. **Recent event, relative to the main event**. This includes events occurring at a nearby location and/or within a short-time window around the main event. For instance, “On Saturday, similar infections were found in 30 pigs on a farm in the Huangpu district of Guangzhou.”
  2. **Aggregation of events between a prior date and a recent/current date**. For instance, “According to data from the Council of Agriculture, 94 poultry farms in Taiwan have been infected by avian flu so far this year.” The temporal expression “so far this year” indicates a relationship between the start of the outbreak and the publication date.
  3. **Recent/current epidemiological status of a disease within an area**. For instance, “In recent months, the disease has spread more rapidly and further west, affecting countries that were previously unscathed.”
  4. **Events that will definitively occur in the future**. In general, this category includes the direct consequences of an event, such as control measures that will be taken. For instance, “All pigs in the complex will be killed, and 3 km and 10 km protection and surveillance zones will be installed.”
- **Old event**: This class includes sentences about events that provide a historical context for the main event. Those sentences contain explicit references to old dates, either absolute (“In 2007”) or relative (“Back in days”). This category includes two groups of sentences:
  1. **Old event**. For instance, “The most recent case of the disease in the UK came in 2007.”
  2. **Aggregation of events between two past dates**. For instance, “Between 2010 and 2011, South Korea had 155 outbreaks of FMD.”
  3. **Past epidemiological status of a disease within an area**. For instance, “Between 2006 and 2010, BTV serotype 8 reached parts of north-western Europe that had never experienced bluetongue outbreaks previously.”
- **Risk event**: This class includes all sentences referring to hypothetical events. These sentences are generally about an area at risk of introduction or dissemination of a pathogen. This category includes two groups of sentences:
  1. **An unaffected area expressing concern and/or preparedness**. For instance, “Additional outbreaks of African swine fever are likely to occur in China, despite nationwide disease control and prevention efforts.”
  2. **An area with unknown disease status**. For instance, “If the outbreak is verified, all pigs at the feeding station will have to be culled, Miratorg said.”
- **General**: This class includes general information about a disease or pathogen. Conventionally, the sentences describe the disease hosts, its clinical presentation and pathogenicity. For instance, “Bluetongue is a viral disease of ruminants (e. g. cattle, sheep goats, and deer).”
- **Irrelevant**: This class includes sentences that do not contain any epidemiological information. This group includes, for instance, disease-unrelated general facts (“Pig imports from Hungary only represented about 0. 64 per cent of all pork products to the UK in 2017.”) or article news artefacts (“Comments will be moderated.”).

##### Information type

The information type level describes the sentence epidemiological topic. As epidemiological topic, we include the notification of a suspected or confirmed event, the description of a disease in an area (“Descriptive epidemiology” and “Distribution”), preventive or control measures against a disease outbreak (“Preventive and control measures”), an event’s economic and/or political impacts (“Economic and political consequences”), its suspected or confirmed transmission mode (“Transmission pathway”), the expression of concern and/or facts about risk factors (“Concern and risk factors”) and general information about the epidemiology of a pathogen or a disease (“General epidemiology”).

- **Descriptive epidemiology**. This class includes sentences containing the standard epidemiological indicators (e.g. disease, location, hosts, and dates) that describe an event. It includes:
  1. **Epidemiological description of the event**. For instance, “Cases of African swine fever (ASF) have been recorded in Odesa and Mykolaiv regions.”
  2. **Information about the pathogenic agent cause of the event**. For instance, “Results indicated that the birds were infected with a new variety of H5N1 influenza.”
  3. **Clinical signs of the suspected event**. For instance, “The remaining buck appears healthy at this time and is showing no clinical signs associated with the disease.”
- **Distribution**. This class contains sentences giving indications on the presence of a disease in a specific area (i.e. a region, a country). It includes:
  1. **Description of the epidemiological status**. For instance, “In recent months, the disease has spread more rapidly and further west, affecting countries that were previously unscathed.”
  2. **Aggregation of events between a past date and a recent/current date**. For instance, “According to data from the Council of Agriculture, 94 poultry farms in Taiwan have been infected by avian flu so far this year.”
- **Preventive and control measures**. This class includes sentences describing:
  1. **Preventive measures**, i.e. all sanitary and physical actions taken to avoid the introduction of a disease into an unaffected area. For instance, “ASF: France about to end the fencing in the borderland with Belgium.”
  2. **Control measures**, i.e. all sanitary and physical actions taken to eradicate a pathogen once introduced into an area (e.g. vaccination, slaughtering, disinfection, zoning, etc.). For instance, “All the infected animals have been killed, and the area has been disinfected.”
  3. **Instructions/recommendations**, i.e. actions for both preventive and control measures, we include recommendations in this class. For instance, “Hunters, travellers, and transporters are asked to take extra care concerning hygiene.”
- **Transmission pathway**. This class includes the sentences indicating the origin (source) of the disease or the transmission route. For instance, “The authorities suggest that the highly contagious virus might have been spread by a river”.
- **Concern and risk factors**. This class includes sentences indicating a risk of introduction or spread of disease in an area. We include two types of sentences in this group:
  1. **Confirmation of suspicion of one or several risk factors**, i.e. an individual, behavioural and environmental characteristics associated with an increased disease occurrence. For example, “A recent wave of inspections revealed 4,000 different biosecurity violations on farms and Gosvetfitosluzhba warned that this could result in further outbreaks soon.”
  2. **Semantic** **expression of fears or concerns** **regarding**: (i) The hypothetical intrusion of a pathogen into an unaffected area. For instance, “ASF is a real threat to the UK,” she said.” (ii) The worrying development of a situation. For instance, “Several countries are affected, alarming governments and pig farmers due to the pace at which the disease has spread.”

- **Economic and political consequences**. This category includes all references to direct or indirect economic or political impacts of an outbreak on an area. It includes the consequences of preventive and control measures. For instance, “Gorod estimated that financial losses due to ASF could amount to 17 million euros to Latvia’s industry in 2017.”
- **General epidemiology**. This category is only used for the sentences labelled “General” as the event type level. It merges the classes “Event description” and “Transmission pathway” described above. In this particular event type level, those two categories include the description of a disease’s hosts, pathogenicity and transmission route. For instance, “The virus is transmitted by midge bites, and it does not affect humans.”

##### Multi-topic sentences

To handle multi-topic sentences, we provide two rules to help annotators make choices:

- If one category (label) is the consequence of another one, the annotator should select the first one. For instance, if a sentence describes both a control measure and its economic effects, the sentence should be labelled as “Preventive and control measures”.
- Both “Concern and risk factors” and “Transmission pathway” provide highly valuable information to assess the risk of emergence or spread of a disease. The annotator should therefore prioritise them against other labels into a multi-topic sentence.

Table 2 provides examples of frequently encountered multi-topic cases and the choice of the main label according to the two rules shown above.

**Table 2:** Resolution of multi-topic sentences in typical cases.

Sentence topics	Example	Possible labels → main label	Rationale
Description of an event and its control measures.	*The Philippines confirms African swine fever, culls 7000 pigs*.	DE, PCM → DE	Control measures are consequences of the event.
Sanitary bans.	*Russia*’*s agriculture authorities introduced temporary restrictions on pig and pork imports from Hungary due to an outbreak of the disease*.	PCM, EPC → PCM	Economic consequences of the ban.
Description of an event and its source.	*The strain detected in China is similar to the one that infected pigs in eastern Russia last year, but there is no conclusive evidence of the outbreak*’*s source, it said*.	DE, TP → TP	Transmission pathway category prevails over the other types.

DE: Descriptive epidemiology, PCM: Preventive and control measures, EPC: Economic and political consequences, TP: Transmission pathway.

#### Annotation agreement

In this section, we describe changes in the agreement metrics during the framework elaboration. As quantitative agreement measures, we calculated the inter-annotation agreement and Cohen’s kappa coefficient. For inter-annotation agreement, we defined three different levels, i.e. total agreement (all annotators reached a consensus), partial agreement (two annotators agreed), and complete disagreement (all annotators disagreed). In the case of multi-labels, we defined the agreement as strict, i.e. there is an agreement between two annotators if they give precisely the same labels.

Cohen’s kappa coefficient (*κ*) is a widely used statistical measure of inter-annotator agreement, which takes into account the extent of agreement expected by chance^15^. *κ* was calculated as follows:

1 $$\kappa =\frac{Pr(a)-Pr(e)}{1-Pr(e)}$$

Where *Pr*(*a*) is the observed agreement among two annotators, *Pr*(*e*) is the hypothetical probability of reaching an agreement.

Table 3 compares the agreement results obtained in step 1 (initial version of the guidelines) and step 3 (the final version of the guidelines). We calculated the kappa by pairs of annotators separately and then computed the average. At step 1, we obtained poor agreement for event type annotations (*κ* = 0.30), while we obtained fair agreement for information type (*κ* = 0.53). Annotators totally agreed on event type labels for only 29% of sentences, while 49% of the sentences obtained a total agreement for information type.

**Table 3:** Agreement statistics at step 1 (initial guidelines, N = 132 sentences) and step 3 (final guidelines, N = 83 sentences).

		Inter-annotator agreement			
		Total agreements	Partial agreements	Disagreements	*κ*
Step 1	Event type	29%	48%	23%	0.30
	Information type	49%	43%	8%	0.53
Step 3	Event type	87%	19%	4%	0.71
	Information type	75%	22%	3%	0.78

The inter-annotator agreement was computed in terms of relative agreements (total and partial), disagreements and Cohen’s kappa (*κ*).

Statistics at step 3 (final guidelines) indicate a substantial improvement in the agreement for both classes. The event type kappa was still lower than the information type kappa (0.71 and 0.78, respectively).

## Discussion

In this Section, we present critical issues that emerged during the framework elaboration process, while outlining our choices to improve the inter-annotator agreement. We first discuss two characteristics of our global framework and then explain two different strategies adopted to modify the annotation guidelines.

### Global framework

#### Double-level annotation

Similar to event annotation approaches in which the annotator labels the event type and its attributes separately^2^, our final annotation framework relies on the attribution of two labels per sentence: event type and information type. We chose this approach because the thematic labels (information type) encompass different temporal and event levels. Their relevance from an event-based surveillance viewpoint differs. For instance, a sentence describing an outbreak that occurred 2 years before the publication date (“Old event”) is obviously less relevant than a sentence describing a current one. However, the type of information provided (description of an outbreak) remains the same. Therefore, the double-level approach is geared toward assigning consistent information type labels among different event statuses. This choice increases the annotation time and complexity, but we believe that it substantially enhances the value of assigned labels by allowing us to consider spatiotemporal and topic labels separately.

#### Single-label annotation

We chose the sentence-based approach to address the lack of granularity in document-level approaches. However, a single sentence may also contain distinct topics. Therefore, until step 3, we allowed multi-labelling (the annotator could allocate as many labels as wished to a sentence, for both event type and information type). For event type, only two sentences from the third dataset had multi-labels, both of them with “Current event” and “Old event”. In both sentences, the reference to historical outbreaks was provided as context, e.g. “It has not been confirmed what caused the outbreak, but there have been other incidents in the region during the 20th century.”

Multi-labelled sentences were more frequent for information type, representing from 14% (12/83) to 34% (28/83) of the sentences according to the annotator. The most frequent associations were:

- “Preventive and control measures” with either “Descriptive epidemiology” or “Economic and political consequences”. In these sentences, there was a causal relationship between the two labels. For instance, in the following sentence, a ban was decided in response to a related outbreak: “The Polish news agency reported that the ban was in relation to two cases of African swine fever found in dead wild boar on the Polish border with Belarus.” These cases were resolved by providing rules to choose the main label in case of a causal relationship. We prioritised the causal label, claiming that it usually contains the main information. In the previous sentence, the outbreak occurrence prevails over the ban. Therefore, the sentence should be labelled as “Descriptive epidemiology”.
- “Descriptive epidemiology” and “Clinical presentation”, mainly referring to mortality (“Two pigs in a population of 36 were found infected - one had died”), or to asymptomatic cases (“Hence affected flocks were detected under routine monitoring as there are no clinical signs associated with the event”). These cases were resolved by merging the two classes, as discussed in section “Merging of classes”.

### Strategies to improve inter-annotator agreement

#### Creation of new classes

During this process, we created the new label “Distribution”. In the first guidelines, sentences such as “In recent months, the disease has spread more rapidly” were labelled by annotators as either “Descriptive epidemiology” or “General epidemiology”. Such sentences describe the current situation but they do not inform on a specific event. On the other hand, they describe an epidemiological situation that depends on a specific context (spatiotemporally locatable). Therefore, they cannot be considered as “General epidemiology”.

#### Merging of classes

We merged the following categories in the annotation process:

1. **Current event and related event**

Initially, we had divided event type labels into three groups for current and past events:

- Current event, i.e. the main event notified in the news article and which recently occurred,
- Related events, i.e. events that happened in the past but are related to the current one,
- Old events, i.e. events that occurred in the past without any link with the current situation (same definition as in the final guidelines)

This distinction between present and related events was the leading cause of disagreements in step 1. Deciding whether an event was a present or a related one was not trivial because it depended on a spatio-temporal cutoff which differed between annotators. Therefore, we decided to gather current and recent outbreaks in the same category (“Current event”). Some authors have proposed using a temporally fixed window. For instance, events occurring within a 3-months window are related^16^. This threshold was also used to label events as historical (occurred more than 3 months ago), in addition to recent events (occurred between 2 weeks and 3 months ago), and present ones (occurred within the last 2 weeks) as described by^2^.

We believe that setting a rigid time window is not consistent with the epidemiological specificity of each disease. We instead decided to aggregate these two categories and distinguish only current/related events from old events. This distinction improved the agreement for the event type level: all six sentences labelled as “Old event” obtained total agreement. In these sentences, typical semantic clues (e.g. the use of temporal expressions such as “back in days” or “in 2006”) explicitly indicated the absence of an epidemiological link.

(2). **Clinical presentation and Descriptive epidemiology**
  The “Clinical presentation” category was present in the first version of the guidelines. The label was mainly used by one annotator in association with the “Descriptive epidemiology” label. It appeared that in these sentences, all symptom-like terms were related to “deaths” or “died”, e.g. “So far, six adult cattle and two calves have died from the disease”. Rather than providing a clinical picture, these expressions were used to indicate the number of cases. We, therefore, decided to merge it with “Descriptive epidemiology” in the final framework.
(3). **Preventive and control measures**

In the intermediate guidelines, we divided preventive and control measures into two distinct categories. This choice increased the number of disagreements in this class because several types of measures could be considered as both preventive or control according to the context. For instance, the slaughtering of infected animals is a control measure for the concerned affected area but is a preventive measure from the unaffected area standpoint (limiting the disease spread). The ban of animal movements, as well as vaccination, can also be control measures (avoid disease spread from the affected area) as well as a preventive measure (prevent disease introduction in an unaffected area). In the BioCaster ontology scheme, this context-dependency made the “control” category the most challenging class in terms of agreement^17^.

### Limitations

Several limitations in the proposed annotation framework should be noted, as they may influence the performance of further classification tasks.

First, we adopted a single-label approach for each level. Not allowing multiple labels per sentence was questionable since several sentences belonged to several classes, and the annotator may have had difficulty in determining which category should take precedence. This may lead to misclassification errors and information loss during the supervised approach. However, the use of multi-labelling raises the issue of finding suitable agreement metrics while adding a major complication in finding proper classification methods^18^. As some typical cases occurred, we tried to harmonise the annotators’ choices by resolving multi-label cases in the guidelines.

Besides, we did not include polarity or sentiment analysis in our labelling scheme. For instance, sentences indicating the absence of outbreaks or a negative result for a test should be labelled as “Descriptive epidemiology”. In practice, sentences claiming a negative event are quite rare in online news narratives. The current frame could be enhanced by adding a polarity label to each sentence as it is necessary to include negation detection to avoid false alarms.

In this Section, we proposed a sentence-based annotation scheme with the aim of going beyond conventional document-based classification and entity recognition. We built the framework by heavily relying on domain expert opinions while intending to find a trade-off between fair inter-annotator agreement and class granularity. The final inter-annotator scores were 0.71 Kappa on average for event type labels and 0.78 Kappa on average for information type labels. While some classes of interest from an epidemiological viewpoint (e.g. “Concern and risk factors”, “Transmission pathway”) are under-represented, we believe that the proposed framework helps increase the number of instances quickly and reproducibility.

## Data Records

The dataset in the CIRAD Dataverse^12^ contains two files, an annotated corpus and the annotation guidelines providing a detailed description of each category. The annotated corpus file contains 1,244 manually annotated sentences extracted from 88 animal disease-related news articles. These news articles were obtained from the database of an event-based biosurveillance system dedicated to animal health surveillance, PADI-web (https://padi-web.cirad.fr/en/). The file is divided into three sheets:

- The first sheet provides metadata about the news articles (the unique id of a news article, its title, the name of the news article website, its publication date and its URL.
- The second sheet contains 486 sentences (from 32 news articles - 10,247 words) which were used to build the annotation framework. Each sentence label corresponds to a consensus label between two or three annotators. Each row corresponds to a sentence from a news article and has two distinct labels, event type and information type. The set of columns contains the id of the news article to which the sentence belongs, the unique id of the sentence, indicating its position in the news content (integer ranging from 1 to *n*, *n* being the total number of sentences in the news article), the sentence textual content, the event type label and the information type label.
- The third sheet contains 758 additional sentences (from 56 news articles - 16,417 words) annotated by a single annotator based on the same annotation framework. The set of columns is similar to the previous sheet.

## Technical Validation

**Fig. 4:**
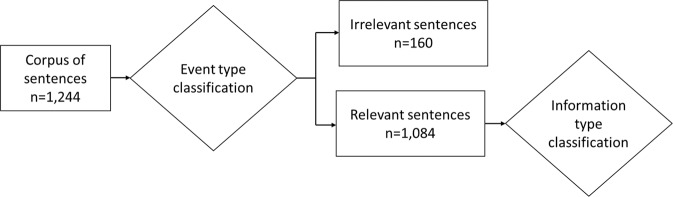
Classification tasks.

We evaluated the value of our annotation approach through a supervised classification task. The classification is called supervised when models are trained on instances whose labels are known (i.e. annotated by domain experts)^19^. The two annotation levels form two consecutive classification tasks: (i) classification of the event type and (ii) topic classification of the information type (Fig. 4). To evaluate the classification on sufficient class sizes, we used both the sentences annotated by two annotators and the additional corpus annotated by a single annotator (section Corpus) and we trained several classifiers (section Classification).

### Corpus

We obtained a final corpus containing 1,244 sentences, among which 160 sentences were irrelevant. The subset of sentences for information type classification hence consisted of 1,084 sentences.

For the event type-level, 64% of the sentences (799/1244) were labelled as “Current event”, 11% (136/1244) as “General”, 8% (105/1244) as “Risk event”, and 4% (44/1244) as “Old event”. “Irrelevant” sentences represented 13% of the corpus (160/1244). The information type-level contained 1084 annotated sentences. Among these sentences, 37% of the sentences (401/1084) were labelled as “Descriptive epidemiology”, 29% (310/1084) as “Preventive and control measures”, 10% (110/1084) as “Concern and risk factors”, 10% (109/1084) as “General epidemiology”, 6% (69/1084) as “Transmission pathway”, 5% (58/1084) as “Economic and political consequences”, and 2% (27/1084) as “Distribution”.

The distribution of sentences at the event type level was highly imbalanced, indicating that disease-related news articles primarily provide information about the current situation (Current event).

The information type level was more balanced, with two classes (“Descriptive epidemiology” and “Preventive and control measures”) representing 67% of the sentences (711/1084).

Even if still modest by its size, our corpus is highly specialised regarding both its domain (i.e. animal health) and its nature (i.e. online news articles). So this corpus type is more specific than the benchmark corpus traditionally used in state-of-the-art approaches in the biomedical NLP domain^20^.

### Classification

The transformation of a corpus of documents into a machine learning-readable format involves two steps. Each document is first transformed into a vector of selected features. Bag-of-words (BOW) is one of the most popular models used to convert textual documents into vectors. In this model, the vocabulary corresponds to all of the terms present in the whole corpus^21^. Each document *d* is encoded in an *n*-dimensional vector where each component *w*_*td*_ represents the absence or presence of a feature (term) *t* in the document (where *n* is the length of the vocabulary). If the feature *t* occurs in the document, the feature weight *w*_*td*_ has a non-zero value.

In a second step, a weight is assigned to each feature in the document. Term Frequency-Inverse Document Frequency (*TF*–*IDF*) is the product of term frequency and inverse document frequency^22^. Terms with the highest *TF*–*IDF* values are distinctively frequent in a document in comparison to the collection of documents.

In this evaluation, each sentence from the corpus represents a document. We simplified the vocabulary by removing punctuation and converting words to lowercase. Then, we transformed all the sentences into the bag-of-words model, using the *TF*–*IDF* weight.

We compared three classifiers that are widely used for text classification:

1. Naive Bayes (NB), is a family of probabilistic classifiers based on Bayes’ theorem. These classifiers are based on the assumption that there is high independence between features. We used a multinomial Naive Bayes classifier, which assumes that features have a multinomial distribution.
2. Support Vector Machines (SVM) is a non-probabilistic and linear classification technique. SVM has been widely used for text classification, including small-sized texts such as sentences^8,23^ and tweets^24^. It achieves robust performance regarding important textual data vector properties, which are sparse and dense (containing few relevant features)^25^. We used a linear kernel parameter (linear SVM) classifier, as linear kernels perform well with textual data^26,27^.
3. Multilayer Perceptron (MLP) is an Artificial neural network-type (ANN) classifier. ANN classifiers were shown to perform well when combined with word embedding representations^28,29^.

We estimated the performances of the trained models via the widely used cross-validation method. We used a fold number of 5, as this value was empirically shown to yield test error rate estimates with low variance, while not being hampered by excessively high bias^30^.

At each fold, we computed the traditional metrics used in supervised classification, i.e. precision, recall, accuracy and F-measure. At the class *A* level, precision corresponds to the proportion of correct sentences classified in class *A* (Eq. 2), and recall corresponds to the proportion of sentences belonging to class *A* that are correctly identified (Eq. 3):

2 $$Precision(A)=\frac{number\;of\;sentences\;correctly\;attributed\;to\;class\;A}{number\;of\;sentences\;attributed\;to\;class\;A}$$

3 $$Recall(A)=\frac{number\;of\;sentences\;correctly\;attributed\;to\;class\;A}{total\;number\;of\;sentences\;belonging\;to\;class\;A}$$

F-measure is the harmonic mean of precision and recall (Eq. 4):

4 $$F-measure(A)=\frac{2\times Precision(A)\times Recall(A)}{Precision(A)+Recall(A)}$$

To calculate the performances over all classes to account for class imbalance, we computed the weighted precision, recall, and F-measure (averaging the frequency-weighted mean per label).

For instance, considering a binary classification between a class A (frequency = *N*_*a*_) and a class B (frequency = *N*_*b*_), the weighted precision *Precision*_*w*_ is:

5 $$Precisio{n}_{w}=\frac{{N}_{a}}{{N}_{a}+{N}_{b}}\times Precision(A)+\frac{{N}_{b}}{{N}_{a}+{N}_{b}}\times Precision(B)$$

Note that the weighted F-measure is not calculated between general values of precision and recall.

The accuracy corresponds to the proportion of correct predictions over the total predictions.

In these experiments, we compared the different classifiers for both event type and information type classification. The performances are summarised in Table 4. MLP and SVM achieved comparatively equal performances and clearly outperformed the NB classifier. These behaviours were identical for event type and information type classification. Classification performances were lower on average for the information type level than for the event type level. Other results (e.g. results by category) are presented in^13^.

**Table 4:** Performances of classifiers trained on bag-of-words (BOW) representations, in terms of weighted precision, recall, F-measure and accuracy over 5-fold cross-validation.

Level	Classifier	Precision	Recall	F-measure	Accuracy
**Event type**	SVM	0.71	0.71	0.70	0.71
		(+/−0.02)	(+/−0.01)	(+/−0.01)	(+/−0.01)
	NB	0.50	0.72	0.55	0.50
		(+/−0.02)	(+/−0.02)	(+/−0.03)	(+/−0.02)
	MLP	0.72	0.70	0.69	0.72
		(+/−0.02)	(+/−0.02)	(+/−0.02)	(+/−0.02)
**Information type**	SVM	0.67	0.66	0.66	0.66
		(+/−0.04)	(+/−0.03)	(+/−0.03)	(+/−0.03)
	NB	0.55	0.67	0.58	0.55
		(+/−0.06)	(+/−0.04)	(+/−0.05)	(+/−0.06)
	MLP	0.66	0.66	0.66	0.65
		(+/−0.03)	(+/−0.04)	(+/−0.03)	(+/−0.03)

## Data Availability

The labelled corpus in our experiments are publicly available in a Dataverse repository: 10.18167/DVN1/YGAKNB^12^. The whole classification and evaluation pipeline was performed using the scikit-learn library (Python): https://scikit-learn.org/^31^.
